# Trapezius muscle transfer for external shoulder rotation: anatomical study

**DOI:** 10.1590/1413-78522014220600931

**Published:** 2014

**Authors:** Mauro Emilio Conforto Gracitelli, Jorge Henrique Assunção, Eduardo Angeli Malavolta, Daniel Takashi Sakane, Marcelo Rosa de Rezende, Arnaldo Amado Ferreira

**Affiliations:** 1. Universidade de São Paulo, Faculdade de Medicina, Institute of Orthopedics and Traumatology, São Paulo, SP, Brazil, Institute of Orthopedics and Traumatology, Faculdade de Medicina da Universidade de São Paulo, São Paulo, SP, Brazil

**Keywords:** Brachial plexus/injuiries, Tendon transfer, Shoulder, Paralysis

## Abstract

**OBJECTIVE:**

To compare the viability of transferring the lower and transverse trapezius to the greater tuberosity using three different techniques.

**METHODS:**

Twelve shoulders from six cadavers were used. The primary outcome was to assess the suture viability of the trapezius muscle transfer to the greater tuberosity in the insertion topography of the infraspinatus, with the arm adducted during internal rotation (hand on the abdomen) and maximum scapular retraction. Three transfers were applied to each shoulder: the lower and transverse trapezius distal insertion (Group 1); lower trapezius alone (Group 2); and lower trapezius insertion and origin (Group 3). Accessory nerve integrity was assessed before and after transfers.

**RESULTS:**

Sutures were viable in 42% (5/12) and 58% (7/12) on Groups 1 and 3, respectively, with no statistically significant difference (Fisher's test, p=0.558); Group 3 exhibited frequent neurologic injury (11/12). Group 2 was the least successful; the tendon did not reach the greater tuberosity, and no sutures were viable.

**CONCLUSION:**

Groups 1 and 3 exhibited the best nongrafting suture viability to the greater tuberosity; however, Group 3 was associated to frequent spinal accessory nerve injury. **Level of Evidence IV, Anatomical Study**

## INTRODUCTION

The external rotation of the shoulder is an essential movement to daily activities with upper limbs and its limitation causes major functional impairment.^1^ Causes of decreased external rotation are injuries of the brachial plexus (traumatic or obstetric) and extensive rupture of the rotator cuff.^1^^,^ 2 In extensive and irreparable rotator cuff tears with limitation of external rotation, the transfer of the latissimus dorsi tendon is most often used in patients younger than 65 years old with no signs of degenerative changes of the glenohumeral joint, with good results for elevation gain and decreased pain.^3^^-^ 6

However, the transfer of the latissimus dorsi has limitations. The action vector of the muscle is not similar to the infraspinatus and the gain of external rotation is limited.^4^ In the presence of subscapularis injuries, the transfer can lead to shoulder subluxation, pain and functional limitation; therefore, it is contraindicated as a single treatment.^7^

An alternative to the latissimus dorsi is the transfer of the lower trapezius, described for cases of obstetrics paralysis^8 ^and braquial plexus injuries.^9^ The action vector of the lower portion of the trapezius is closest to the infraspinatus muscle and the results on gain of external rotation are promising.^8^^,^ 10 ^,^ 11 However, its distal reach in the greater tuberosity has not been studied previously, and implies the need for tendon grafting and immobilization in abduction and external rotation.^9^ The association of transferring the origin of the lower portion of the trapezoid could increase the distal reach of the insertion and has not been previously described in the literature.

The aim of this study is to describe in cadavers the anatomic parameters and the feasibility of three techniques of trapeze transfer: lower portion together with the transverse, lower portion in isolation and double transfer of the lower portion (origin and insertion).

## METHODS

This study was approved by the Ethics Committee of our Institution under number 949. Twelve shoulders from six randomly selected fresh-tissue cadavers were used.

Three different techniques were performed on each shoulder. Sequentially and always in the same order we performed transfer of the lower portion combined with the transverse (Group 1), transfer the lower portion alone (Group 2) and transfer of the origin and insertion of the lower portion (Group 3). After each transfer the anatomical parameters were evaluated.

### Surgical technique 

The cadavers were prone positioned, a wide posterior approach being conducted.

**Figure 1:**
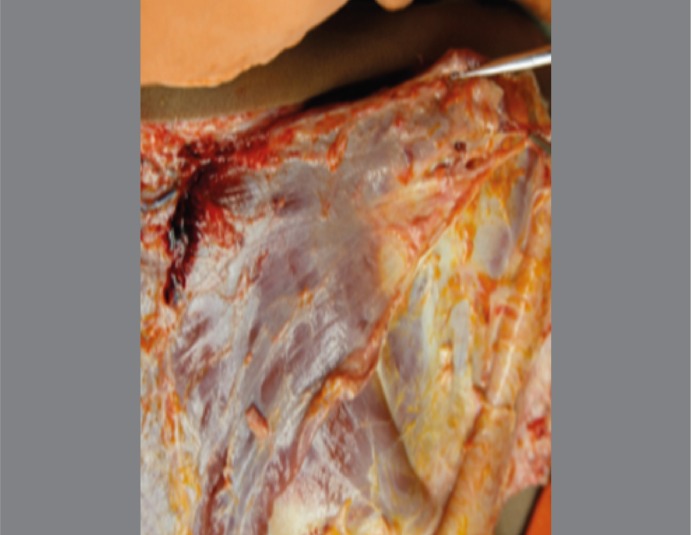
transfer of the lower portion combined with the transverse trapezius muscle (Group 1).

For Group 1, the lower portion together with the transverse portion of the trapezius was isolated, separated from its upper portion on the spine of the scapula. (Figure 1) The insertion was resected together with the distal aponeurosis of the trapezius, without including the deltoid fascial tissue or bone fragments. The posterolateral corner of the acromion was used as the site of lateral resection parameter. The tendon was repaired using continuous non-absorbable suture, and the muscle was raised separated from the infraspinatus, deltoid and rhomboid.

The lower level of transfer was determined by the lower border of the scapula. The accessory nerve was identified and marked, as well as other landmarks described below, and the origin of the deltoid on the scapular spine was released. The viability of the transfer to the greater tuberosity was assessed and rated positive when the tendon reached the posterior aspect in the insertion topography of the infraspinatus.

**Figure 2:**
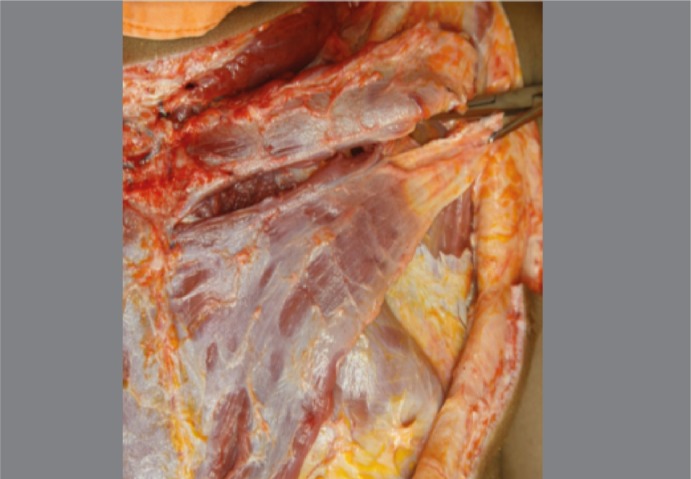
The lower portion of the trapezius was separated from the transverse portion and tranferred alone (Group 2).

**Figure 3:**
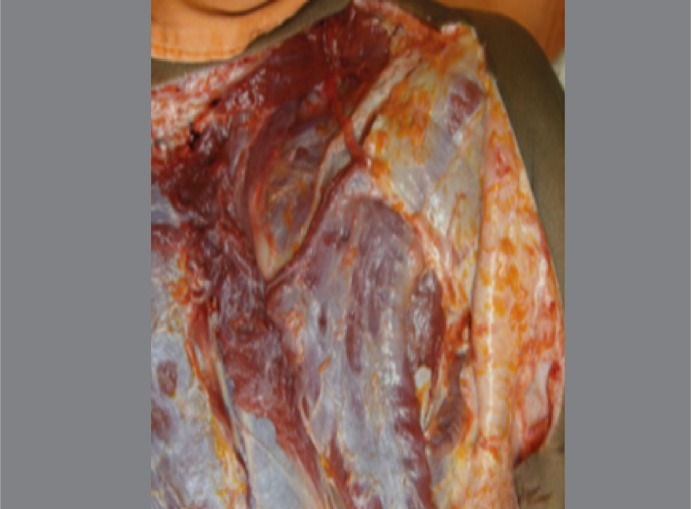
The origin of the lower portion of the trapezius muscle was released and transferred to the medial border of the scapula (Group 3).

The lower trapezius of Group 2 was detached from the transverse portion and transferred alone. (Figure 2) Finally, the origin of the lower trapezius next to the spinous processes was released in Group 3 and then attached to the medial scapular margin using transosseous sutures. (Figure 3) The stages involving distal portion repair, soft-tissue dissection, and greater tuberosity transfer viability assessment were performed similarly for all groups.

### Outcomes and anatomic variables 

Primary outcome: Transfer viability with the arm adducted during internal rotation (hand on the abdomen) and maximum scapular retraction.

Secondary outcomes: Transfer viability with the arm adducted during internal rotation (hand on the abdomen) and maximum scapular protraction.

Anatomical parameters: The anatomical parameters assessed before transfers and measured with maximum protraction of the scapula are presented in Table 1.

**Table 1:** Anatomical parameters assessed.

Anatomical parameters
Width of the scapula (from the superior medial angle to the superior-lateral scapular spine)
Height of the scapula (from the inferior to the superior-medial angle)
Distance from the lateral insertion of the transverse portion of the trapezius (TPT) to the superolateral angle of the scapula (SLAS), measured towards the spine of the scapula
Distance from the lateral insertion of the lower portion of the trapezius (LPT) to the superolateral angle of the scapula (SLAS), measured towards the spine of the scapula
Distance of the spinous processes of the spine to the greater tuberosity of the humerus (GTH), measured towards the scapular spine
Length of transverse and lower portion of the trapezius, from its medial origin to its lateral insertion
Length of lower portion of the trapezius, from its medial origin to its lateral insertion
Length of the fascia and tendon of the transverse portion and lower trapezius
Tendon width of the transverse and lower portion at 20 mm from its insertion
Height of the origin of the lower portion of the trapezius (scapular spine to its lower limit)
Distance from the vascular-nerve bundle at the medial edge of the scapular spine

The integrity of the accessory spinal nerve was assessed before and after performing the three tendon transfers.

### Statistical analysis 

An a priori sample size was calculated based on 80% power and 5% Type I error. The study hypothesis is that the technique of Groups 2 and 3 will be viable for suture around 30% of the sample and the technique of Group 1 in at least 90%. Thus, we determined that 10 shoulders will be needed. For this calculation the online calculator www.stattool.net was used.

Data normality was tested using the Shapiro-Wilk test and homogeneity of the groups using the Levene test.

Categorical and continuous variables were displayed as means and standard deviations (parametric data) or median and percentiles (nonparametric data). For the primary and secondary (correlation between viability of the various suture techniques) outcomes, the Chi-square test (for categorical data) was used. Software SPSS 19.0 for Windows was used for analysis.

## RESULTS

Five cadavers were male, and one was female. Their mean age, height, and weight were 63 ± 11.77 years old, 165.66 ± 4.69 cm, and 56.2 ± 5.53 kg, respectively.

The anatomical parameters measured before the tendon transfers at maximum protraction of the scapula are presented in

Table 2. The spinal accessory nerve and the associated vascular pedicle was located on average 3.25 ± 1.63 cm medial to the medial border of the scapular spine.

**Table 2:** Results of the measurements of the anatomical parameters assessed before transfers

Anatomical parameters	Mean Standard deviation
Width of the scapula	13.66 ± 1.21 cm
Height of the scapula	15.83 ± 1.13 cm
Distance from the lateral insertion of TPT to SLAS	4.13 ± 1.49 cm
Distance from the lateral insertion of LPT to SLAS	9.25 ± 0.73 cm
Distance from the spine to GTH	25.21 ± 2.29 cm
Lenght of LPT + TPT (muscle and tendon)	20.04 ± 2.57 cm
Lenght of LPT (muscle and tendon)	18.38 ± 4.06 cm
Length of TPT tendon	8.22 ± 2.92 cm
Length of LPT tendon	6.21 ± 0.96 cm
Width of TPT tendon	1.10 ± 0.32 cm
Width of LPT tendon	2.40 ± 0.53 cm
Height of LPT origin	14.75 ± 2.33 cm

For the primary endpoint of the study, the feasibility or nor of the transfer, with the upper limb adducted and internally rotated, with the scapula at full retraction, we obtained in Group 1 (transfer of the distal insertion of the lower and transverse portion of the trapezius), the feasibility of suture in 42% of cases (5/12). In Group 2 (transfer only of the lower portion of the trapezius), the tendon has not reached the greater tubercle in any case. Group 3 (transfer from the origin and insertion of the lower portion of the trapezoid) suture was possible in 58% of cases (7/12). When comparing Group 1 with Group 3 no statistically significant difference was found regarding the feasibility of the transfer (Fisher test, p = 0.558). It can be seen that Group 2 obtained no viability in all cases, being the worst technique of choice for this procedure without the use of tendon graft.

When we evaluate the feasibility of the transfer, with the upper limb adducted and internally rotated, with the scapula in maximum protection, in Group 1, the transfer was feasible in 17% (2/12 shoulders). In Group 2, the tendon has not reached the greater tuberosity in any case. In Group 3, the suture was possible in 42% of cases (5/12 shoulders). When comparing Group 1 with Group 3 no statistically significant difference (Fisher test, p = 0.47) was found.

Regarding the integrity of the spinal accessory nerve after completion of muscle transfers, we observed that after the surgical procedures of groups 1 and 2 we found nerve integrity in all cases, but after carrying out transfers on Group 3, we found a high rate of injury to this nerve (11/12 shoulders).

## DISCUSSION

Muscle transfers are widely used to restore shoulder function in patients with obstetric palsy, brachial plexus injuries and irreparable rotator cuff tears. For increased strength and range of motion for external rotation, it is described the transfer of latissimus dorsi and/or teres major muscle to the superolateral region of the humeral head or lateral cortex of the proximal humerus, with reasonable results on external rotation gain.^4^^-^ 6 ^,^ 12 ^,^ 13

Some surgeons believe that the transfer of these tendons to the rotator cuff contributes only to stabilize the humeral head, through the tenodesis effect,^4^^,^ 14 because these tendons are strong internal rotators and have a small phasic conversion after the transfer and their action vector differs from that of external shoulder rotators.^4^^,^ 15

Furthermore, in many cases of obstetric paralysis or traumatic injury of brachial plexus in adults, innervation of these muscles was also injured and they are not available for transfer.^8^^,^ 11

A transfer of the lower trapezius to the infraspinatus insertion was recently described, and it exhibited promising results.^7^^-^ 11 ^,^ 16 This transfer has the advantage, relative to the latissimus dorsi and teres major muscles, an action muscle vector closer than the one presented by the external rotators of the shoulder. Hartzer *et al*.^17^ found that at zero degrees abduction, the transfer of the lower trapezoid is potentially more effective in restoring the motion of external rotation regarding the transfer of the latissimus dorsi muscle. Moreover, during the movement of external rotation of the shoulder, the trapezius muscle has a phasic contraction with the infraspinatus and teres minor muscles, facilitating the rehabilitation of patients undergoing this transfer.^8^^,^ 16

However, this transfer does have some limitations; Elhassan *et al*.^11^ needed tendon graft to perform the transfer of the tendon insertion of the lower portion of the trapezius muscle in the greater tuberosity of the humerus. In our work we also failed in every case to obtain this type of transfer without using tendon graft (Group 2). This finding is justified by the great distance of the supero-lateral angle of the scapula (SLAS) regarding the inclusion of the lower portion of the trapezius muscle (LPT), on average 9.25 ± 0.73 cm.

Bertelli 8 ^,^ 10 performed ​​the transfer of the lower trapezius insertion, extending the tendon to the fascia overlying the spine of the scapula to the acromion. In addition, he does not make the suture of the transfer in the greater tuberosity, but held it in the infraspinatus tendon with the upper limb in maximum external rotation. The transfer by this author is very similar to our Group 1, where we obtained 17% of the possible transfers without the use of grafts with maximum scapular protraction and in 42% with the scapula at full retraction. With external rotation and/or abduction of the upper limb, we would probably have a greater number of possible transfers without using the tendon graft.

The present study described a novel technique of transferring the lower trapezius muscle to the external rotators of the shoulder, with the release of muscular origin of the spinous processes and their suture on the medial border of the scapula and suture of the insertion of the trapezius muscle in the lower greater tuberosity of the humerus (Group 3). In this group, we found higher suture viability, 58% with the scapula retracted and 42% with protracted scapula. However, when comparing Groups 1 and 3 we did not find statistically significant difference regarding the feasibility of the transfer, regardless of the position of the scapula. Due to the limited number of shoulders on which we performed the tendon transfers, such lack of difference may represent a type II error.

Despite allowing muscle transfer without the use of tendon graft, we found a high rate of injury or excessive tensioning of the spinal accessory nerve (11/12 shoulders) with the technique represented in Group 3. Probably, to make this transfer would require an additional dissection the vasculo-nervous for greater mobilization and avoid its injury.

This study has some limitations. Due to stiffness of corpses, we were unable to test the feasibility of muscle transfers with upper limb in external rotation and abduction, what would potentially increase the likelihood of the lower trapezius muscle tendon and/or transverse to reach the greater tuberosity of the humerus. We also could not observe whether the muscle transfers would lead to some muscle ischemia by compression of the vascular pedicle.

We could not determine the appropriate muscle tension to hold the muscle transfer and evaluate its effect on glenohumeral joint movement, since it is an anatomical cadaver study. There are some clinical studies on this topic, demonstrating a considerable increase in the range of external glenohumeral rotation.^7^^-^ 11

The transfers of the insertion of the lower and transverse portions of the trapezius were viable in 42% of the cases when the scapula was in retraction; therefore, this technique might represent an alternative to using grafts or allow the use of shorter tendon grafts. However, our anatomical study showed that the insertion tendon of the transverse portion of the trapezius exhibited 50% of the lower portion tendon width. Biomechanical and clinical tests are needed to investigate transfer resistance as well as its ability to generate the external rotation motion of the glenohumeral joint.

## CONCLUSION

The transfer of the origin and insertion of the lower portion of the trapezius and the transfer of the distal insertion of the lower and transverse portion of the trapezius showed the best results regarding the feasibility of the greater tuberosity suture without the use of tendon grafts. However, the transfer of the origin and insertion of the lower trapezius muscle show a high rate of injury of the spinal accessory nerve.
